# *Candida dubliniensis* Infection, Singapore

**DOI:** 10.3201/eid0804.010423

**Published:** 2002-04

**Authors:** Ai Ling Tan, Grace CY Wang, Yoon Wan Chiu

**Affiliations:** Singapore General Hospital, Singapore

**To the Editor:** We read with interest the letter by Marriott et al. (*1*) describing the first case of *Candida dubliniensis* fungemia in Australia.

We report the first two cases of *C. dubliniensis* infection in Singapore. We have been using the API 20C AUX on all yeast isolates from blood, sterile fluids, and tissue to screen for *C. dubliniensis* since May 2000. To our knowledge, this infection has not been previously reported from a Southeast Asian country.

The first patient was a 49-year-old woman with adult polycystic kidney and liver disease; she had mild chronic renal failure and a past history of nephrotic syndrome. She was admitted to the renal unit on May 9, 2001, for management of ascites. A septic workup showed leukocytosis (34.67 x 10^9^/L) with predominance of neutrophils (95%). Blood culture received on May 31 grew yeast with two slightly different forms, one large and one small. The isolates, investigated separately, were germ-tube positive. The API 20C AUX profile for the larger isolate was 6072114 at 48 hours’ incubation (96.2% certainty for *C. dubliniensis*) and 6072134 for the smaller isolate (99.8% certainty for *C. dubliniensis*). The yeasts grew well on potato dextrose agar at 35°C but poorly at 42°C and 45°C. In addition, electrophoretic karyotyping with pulsed-field gel electrophoresis showed that both isolates had identical patterns, with eight chromosome fragments, one of which was <1 megabase (Mb), indicating that the two morphologically different strains were the same karyotypically. The pattern obtained was in keeping with results obtained by Jabra-Rizk et al. (*2*), with *C. dubliniensis* showing a chromosome-sized band of <1 Mb. For *C. albicans*, by contrast, all bands were >1 Mb. Five control strains—*C. dubliniensis* (RCPA Microbiology QAP item 2001:2:7A), *C. albicans* ATCC 90028, and three clinical strains of *C.* *albicans—*were also run together with the two strains; the results obtained were consistent with those of Jabra-Rizk et al. (*2*). The MIC of the isolates to fluconazole by E-test was 0.75 μg/mL, indicating susceptibility. Disseminated intravascular coagulopathy due to sepsis from a possible ruptured liver cyst developed in the patient. Despite broad-spectrum antibiotics and amphotericin B, hemodialysis, and intensive-care support, she died 5 weeks after admission. Except for *C. dubliniensis* candidemia, *Candida* species isolated from the urine and endotracheal secretions (speciation not done), and *Acinetobacter baumannii* (cultured from the endotracheal secretions and femoral catheter tip), no other important microorganisms were isolated. Peritoneal fluid cultures did not yield any microorganisms.

Soon after the first case, *C. dubliniensis* was isolated from a sputum culture and bronchial alveolar lavage cultures from a 50-year-old Chinese woman, who had myelodysplastic syndrome and was hospitalized for pneumonia. She had been previously treated with chemotherapy and had multiple admissions for infection. The same phenotypic and karyotypic methods as the first patient were used to identify the isolate. The API 20C AUX profile was 2152134 at 48 hours (98.5% certainty for *C. dubliniensis*). The MIC of fluconazole by E-test was also 0.75 μg/mL. Pancytopenia developed, and the patient’s condition deteriorated. She died of pneumonia despite transfusions and treatment with broad-spectrum antibiotics and amphotericin B. Microbiologic investigations for bacteria, tuberculosis, pneumocystis, *Legionella,* and viruses did not yield positive results except for *Corynebacterium* species in the bronchial alveolar lavage fluid.

Although *C. dubliniensis* was first associated with oral candidiasis in HIV-infected persons (*3*), several reports now link the organism to non-HIV patients who were immunosuppressed due to chemotherapy, hematologic malignancy (*4*), and end-stage liver disease (*1*). Our two patients were not HIV positive but were immunosuppressed. In vitro susceptibility results showed that our patients should have responded to the usual antifungal treatment. However, they died despite appropriate therapy.

## References

[1] Marriott D, Laxton M, Harkness J. *Candida dubliniensis* candidemia in Australia. Emerg Infect Dis. 2001;7:479. 11384536 10.3201/eid0703.010326 PMC2631807

[2] Jabra-Rizk MA, Baqui AAMA, Kelley JI, Falkler WA Jr, Merz WG, Meiller TF. Identification of *Candida dubliniensis* in a prospective study of patients in the United States. J Clin Microbiol. 1999;37:321–6. 9889211 10.1128/jcm.37.2.321-326.1999 PMC84296

[3] Sullivan DJ, Westermemg TJ, Haynes KA, Bennett DE, Coleman DC. *Candida dubliniensis* sp. nov.: phenotypic and molecular characterization of a novel species associated with oral candidiasis in HIV-infected individuals. Microbiology. 1995;141:1507–21. 7551019 10.1099/13500872-141-7-1507

[4] Meis JFGM, Ruhnke M, DePauw BE, Odds FC, Siegert W, Verweij PE. *Candida dubliniensis* candidemia in patients with chemotherapy-induced neutropenia and bone marrow transplantation. Emerg Infect Dis. 1999;5:150–3. 10081684 10.3201/eid0501.990119 PMC2627682

